# Stage call: Cardiovascular reactivity to audition stress in musicians

**DOI:** 10.1371/journal.pone.0176023

**Published:** 2017-04-24

**Authors:** Theerasak Chanwimalueang, Lisa Aufegger, Tricia Adjei, David Wasley, Cinzia Cruder, Danilo P. Mandic, Aaron Williamon

**Affiliations:** 1 Department of Electrical and Electronic Engineering, Imperial College London, London, United Kingdom; 2 Centre for Performance Science, Royal College of Music, London, United Kingdom; 3 Faculty of Medicine, Imperial College London, London, United Kingdom; 4 Cardiff School of Sport, Cardiff Metropolitan University, Cardiff, United Kingdom; 5 Department of Research and Development, Conservatory of Southern Switzerland, Lugano, Switzerland; University of Illinois at Urbana-Champaign, UNITED STATES

## Abstract

Auditioning is at the very center of educational and professional life in music and is associated with significant psychophysical demands. Knowledge of how these demands affect cardiovascular responses to psychosocial pressure is essential for developing strategies to both manage stress and understand optimal performance states. To this end, we recorded the electrocardiograms (ECGs) of 16 musicians (11 violinists and 5 flutists) before and during performances in both low- and high-stress conditions: with no audience and in front of an audition panel, respectively. The analysis consisted of the detection of R-peaks in the ECGs to extract heart rate variability (HRV) from the notoriously noisy real-world ECGs. Our data analysis approach spanned both standard (temporal and spectral) and advanced (structural complexity) techniques. The complexity science approaches—namely, multiscale sample entropy and multiscale fuzzy entropy—indicated a statistically significant decrease in structural complexity in HRV from the low- to the high-stress condition and an increase in structural complexity from the pre-performance to performance period, thus confirming the complexity loss theory and a loss in degrees of freedom due to stress. Results from the spectral analyses also suggest that the stress responses in the female participants were more parasympathetically driven than those of the male participants. In conclusion, our findings suggest that interventions to manage stress are best targeted at the sensitive pre-performance period, before an audition begins.

## Introduction

The first attempt to introduce a taxonomy of stress dates back to Hans Selye in 1936, who defined stress as a “non-specific endocrine response” [1]. Current research explains stress through the modulation of the autonomic nervous system (ANS) resulting from physical, environmental, or other psychosocial stressors, where both the sympathetic (SNS) and parasympathetic nervous systems (PNS) are involved in the regulation of functions including heart rhythms, respiration, and blood pressure [2].

Music performance is a particularly apt domain for studying ANS reactivity to stress [3]. The parameters of performance are set by widely shared conventions and by the constraints of the music being performed. This enables close control, documentation, and monitoring of situational variables, while musicians and their audiences interact naturally. Moreover, the sources of stress in music performances are manifold, from executing technically demanding compositions to responding to critical audiences in rarified performance spaces. For professional musicians, the expectation to deliver high-quality performances—no matter where, when, and for whom—has been linked to debilitating and sometimes career-threatening mental and physical distress [3].

The majority of stress research into music performance has focused on the psychological construct of performance anxiety using questionnaires, while neglecting the objective assessment of corresponding physiological components. A notable exception is the work of Craske and Craig [4], who studied 40 musicians in both private and public performance contexts. Heart rate (HR) was extracted as a measure of autonomous body response prior to and after the first note played. State anxiety was assessed using a questionnaire before each performance [5]. The results showed that the highest physiological arousal was captured in the public performance scenario and that the average HR *prior* to each performance was significantly higher than the average HR *during* the corresponding performance. In a similar study, Abel and Larkin [6] recruited 22 musicians to perform in front of a panel of judges. Elevations in measured HR and state anxiety were observed from the baseline to the pre-performance period, with a peak in physiological reactivity detected just before participants stepped onto stage. However, HR was not measured during performance, which prevented direct comparison with the pre-performance period. While research into physiological stress in musicians has usefully examined the degree of cardiovascular reactivity at different points in the performance cycle (e.g. pre-, during-, and post-performance), existing studies have relied mainly on HR [4, 6–8] rather than the dynamically more informative heart rate variability (HRV).

The analysis of HRV in the time, frequency, and non-linear domains is now widely used to assess the biomarkers of stress. In particular, the high frequency (HF) power in HRV is considered to reflect PNS activity influenced by vagal control, while the low frequency (LF) power is multifaceted and was previously believed to reflect SNS activity. The ratio of the power in the LF to HF frequency bands (so-called LF/HF ratio) was long thought to indicate the degree of sympathovagal balance; the higher the ratio, the greater the dominance of SNS, while a lower ratio was thought to suggest the dominance of PNS activity [9, 10]. However, the LF/HF ratio is not a reliable indicator of stress, as the LF band reflects both SNS and PNS activity due to the nonlinear behavior of the vagus nerve [11–16]. Nevertheless, in many psychophysiological stress studies [17–22], stressors have been found to be associated with an increase in the LF band and a decrease in the HF band.

Although the analysis of HRV in the frequency domain can identify and capture changes in stress, nonlinear analysis in the form of structural complexity has recently been used to quantify degrees of determinism versus randomness in signals and has become prevalent [23–26]. This nonlinear metric is conveniently interpreted though the complexity loss hypothesis [27], which asserts that physiological responses in organisms under constraints (illness, ageing, and other inhibitions such as stress) exhibit lower structural complexity (fewer degrees of freedom) than physiological responses in healthy organisms. Among the available entropy measures, the data-driven sample entropy (SampEnt) methods are particularly interesting; low levels of SampEnt indicate a time series of high regularity, while increasing values of SampEnt correspond to a higher degree of irregularity [24]. An improved version of the SampEnt method, called Fuzzy entropy (FuzzEnt), has also been recently employed [25]. However, neither completely deterministic nor purely stochastic data are truly complex, as structural complexity is reflected in long-range correlations.

Most studies of complexity loss in HRV have been conducted in the context of understanding cardiovascular diseases. Our recent study on psychosocial stress in public performance [28] was conducted with a single expert pianist and provided evidence of a reduction in complexity in HRV in response to increased stress levels [28–32]. In particular, the study examined the complexity of the HRV of the performer for the first 20 minutes of low- and high-stress performances, and concluded that (i) complexity of HRV was significantly lower during the high-stress performance and (ii) the SampEnt method exhibited better discrimination between the stress conditions, compared with standard spectral analyses.

The aim of the present study, therefore, is to establish a systematic approach to the examination of physiological stress in music performance contexts. The evolution of stress responses to performance was modeled over a cohort of 16 musicians whose electrocardiograms (ECGs) were recorded for 5 min prior to and 5 min during two performances: (i) a low-stress condition with no audience present and (ii) a high-stress condition in front of an audition panel. An audition was deemed particularly well suited for the high-stress scenario, owing to the scrutiny under which musicians are placed. Auditions also allow enhanced experimental control and maintain high ecological validity through the assignment of appropriate pieces to be played and the possibility of demarcating precise timings before and during performance. We used modern wearable sensing devices for the collection of ECG data and advanced analysis techniques to capture the signature of stress. Specifically, multiscale sample entropy (MSE) [26] and multiscale fuzzy entropy (MFE) [33] approaches were introduced in order to examine entropy values over increasing time scales, thus producing so-called complexity profiles (the MSE and MFE algorithms are described in S1 File). In this way, not only do we account quantitatively for objective aspects of performance stress, but we are also able to identify the critical timing of stress reactivity and estimate the most appropriate period during which to intervene using stress management strategies.

## Material and methods

### Participants

Eleven violinists from the Royal College of Music (RCM) and five flutists from the Conservatory of Southern Switzerland (CSI) participated in the study. The cohort consisted of healthy male (n = 9) and female participants (n = 7) with a mean age of 23.12±2.42 years (range 19–27), all of whom were advanced music students with at least 10 years of public performance experience. Recruitment at the RCM took place from October 2012 to March 2013, with data collection in March 2013, while recruitment at the CSI took place from March to April 2011, followed by data collection in May 2011. Participants were assigned to perform individually the *Allemande* from J. S. Bach’s Partita No. 2 in D minor for solo violin (BWV 1004) or the *Allemande* in A minor for solo flute (BWV 1013).

### Physiological and psychological measures

For the violinists, ECG was recorded using the Bioharness, a physiological monitoring device from Zephyr^TM^ which has been validated in a similar scenario by Johnstone and colleagues [34, 35]. The raw signals were acquired at a sampling rate of 250 Hz. For the flutists, ECG was collected using the PowerLab (model 26T), a similar acquisition device from ADInstruments. Electrodes were attached at the chest and intercostal spaces (between ribs VI and VII). The raw signals were acquired at a sampling rate of 1000 Hz.

### State anxiety

Prior to each performance, participants completed Form Y1 of the State-Trait Anxiety Inventory (STAI) [5], a 20-item questionnaire which assesses the emotional state of a person based on subjective feelings of nervousness. Each item is rated on a 4-point scale (1 = *almost never* to 4 = *almost always*) with cumulative scores ranging from 20 (low anxiety) to 80 (high anxiety). For reference, the moderate-level score among young men is 36.47±10.02, and among young women, it is 38.76±11.95 [5].

### Experimental design

#### Induction session

Before conducting the experiment, every participant attended a 20-minute induction session and confirmed their willingness to deliver multiple polished performances (on separate days) of either the *Allemande* from J. S. Bach’s Partita No. 2 in D minor for solo violin (BWV 1004) or the *Allemande* in A minor for solo flute (BWV 1013). The performance conditions were explained: (1) the low-stress condition involved the assessment of physiological responses during a private performance without any external attendees apart from the researchers managing the measurement; (2) the high-stress condition was a performance in front of an audition panel, composed of three members of staff from the RCM (for violinists) and the CSI (for flutists). The participants were also required to provide background information on their musical experience and general health. On the day of each performance, all participants confirmed that they had not taken anxiolytic medications or other substances that may affect their perceptions and physiological responses to the performance scenarios.

#### Recording protocol

**Fig 1:**
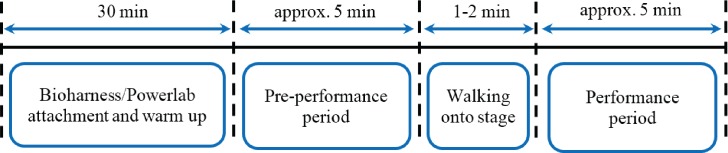
The experimental protocol. The timeline was designed for collecting physiological data from the participants experiencing the low- and high-stress conditions.

The low- and high-stress performances were scheduled on separate days, and the order was counterbalanced across participants. The musicians were asked to arrive 30 minutes before the pre-performance period for the attachment of the ECG recording devices and also for usual performance preparation (e.g. warming-up, tuning, and rehearsing). Stage calls were given at 20 minutes and 10 minutes before performance by a member of the research team acting as the “backstage manager”. At 5 minutes before performance, participants were brought to a backstage area and asked to complete Form Y1 of the STAI. The backstage manager then gave a confirmation signal and allowed the participant to enter the performance room; this period is referred to as *pre-performance* (PP). It is important to note that the pre-performance includes the 2–3 minutes needed to walk into the performance room and settle for the performance. The participants then performed the designated pieces, for approximately five minutes in duration (5.06 ± 0.22 minutes for the violinists and 5.19 ± 0.10 minutes for the flutists); this period is referred to as *performance* (P). Time labels were manually marked by the research team for every condition analyzed. In addition to the ECG recordings, all performances were recorded using a video camera. The experimental protocol is summarized in Fig 1.

### Data analysis

#### Pre-processing

**Fig 2:**
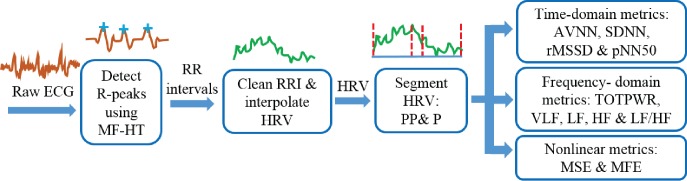
Analysis framework from recorded ECG to the analysis of time, frequency and nonlinear performance metrics. MF-HT stands for the combination of matched filter and Hilbert transform algorithms, while PP and P respectively designate the periods pre- and during performance.

**Fig 3:**
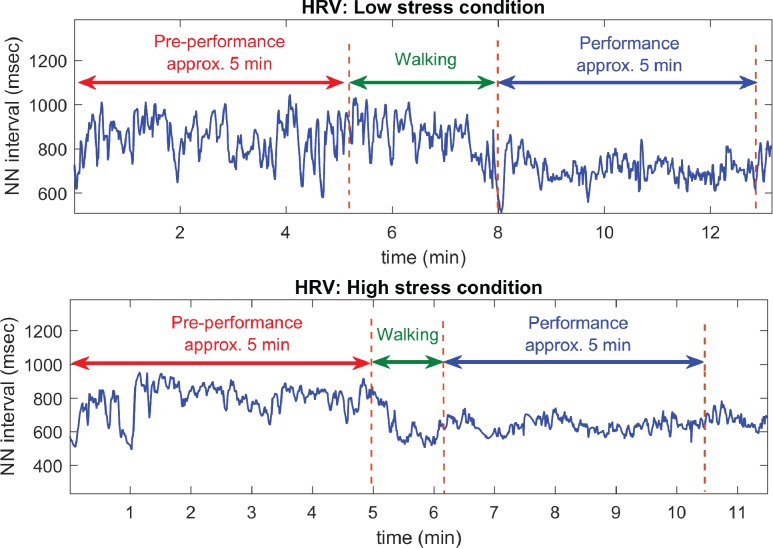
An example of HRV. HRV signal of a participant, interpolated from the NN intervals in the low- and high-stress conditions.

The R-peaks in the recorded ECGs were detected using a combination of matched filtering and Hilbert transform algorithms [36], with the initial QRS complex identified based on a search for ventricular depolarization (QRS) patterns in the segmented windows of ECG data. The RR intervals were then created by subtracting the time-intervals of consecutive R-peaks. However, premature ventricular contractions, or ectopic beats, present in the original RR intervals are known to adversely affect the results from any analyzing metrics [37, 38]. To this end, a custom-made algorithm developed for detecting anomalous peaks in the RR intervals was used to address those erratic behaviors. The detected anomalous beats were then replaced by interpolated data, generated using a smoothing function (the algorithm for detecting HRV anomaly peaks was created by the authors’ team and has not been published; it has been used here under agreement of an unrevealed detail); this resulted in normal sinus to normal sinus intervals (NN intervals). The HRV signals were then generated from the NN intervals using cubic spline interpolation at the sampling frequency of 8 Hz [39]. The HRV signals were segmented into the pre-performance (PP) and performance (P) periods using the time labels created during the experimental setup. The block diagram of the whole analysis framework is shown in Fig 2, and an example of the HRV is shown in Fig 3. Clean HRV data extracted from the recorded ECG of the 16 musicians are available in S2 File.

#### Time domain, frequency domain, and nonlinear analyses

The analyses were conducted in the time, frequency, and nonlinear domains. The averages of NN intervals (AVNN), the standard deviation of all NN intervals (SDNN), the square root of the mean of the squares of the differences between adjacent NN intervals (rMSSD), and the percentage of differences between adjacent NN intervals (pNN50) were used as standard time-domain metrics [40]. For the power spectral density (PSD) analysis, the aggregate PSDs in the low frequency band (LF: 0.04–0.15 Hz) and high frequency band (HF: 0.15–0.4 Hz) were computed using the Welch PSD estimator; the ratio between the power of the LF and HF bands (LF/HF) was calculated as an additional metric. A further power spectral analysis for sex was also conducted, in which the powers within the LF and HF frequency bands were normalized by the power in the 0.04–0.5 Hz band. The 0.04–0.5 Hz band was used in the normalization due to the fact that the physiological explanation of powers below 0.04 Hz in HRV is believed to be questionable [10], and the information content of heart rate signals is contained below 0.5 Hz [41].

For the nonlinear analysis, the MSE and MFE were computed to quantify degrees of uncertainty of HRV over ten scales. The selected parameters for estimating SampEnt were: embedding dimension = 2, tolerance = 0.15 times the standard deviation of the data, time lag = 1 [26], while the optimal parameters used in FuzzyEnt were found empirically: embedding dimension = 2, tolerance = 0.01, time lag = 1, and a second degree Gaussian membership function. The areas of the entropy curves over the whole ten scales (the complexity profile) were considered as the nonlinear metric.

The t-test was used to examine statistical differences in each time, frequency and nonlinear metric according to four possible comparisons: (1) high-stress: pre-performance (PP) vs performance (P), (2) low-stress: pre-performance (PP) vs performance (P), (3) pre-performance (PP): low- vs high-stress, and (4) performance (P): low- vs high-stress (see Table 1). After grouping the participants based on their sex, we also assessed the differences between the LF and HF responses from the PP and P periods. Scores on the state anxiety inventory (STAI-Y1), completed before the low- and high-stress performances, were compared using a paired-samples t-test.

**Table 1:** Statistical tests of frequency and nonlinear metrics.

Metrics	Descriptive statistics					Statistical comparisons			
	Low- stress: PP mean±SD		Low- stress: P mean±SD	High-stress: PP mean±SD	High- stress: P mean±SD	Low- stress: PP vs P t_15_, *p*	High- stress: PP vs P t_15_, *p*	PP: low- vs high- stress t_15_, *p*	P: low- vs high- stress t_15_, *p*
Time-domain									
AVNN	624.0±78.6	637.2±108.2		557.5±91.9	555.3±115.0	-0.52, 0.61	0.07, 0.95	3.00, 0.009	**4.01, 0.001**
SDNN	51.4±20.6	42.4±17.5		48.7±21.6	38.6±18.8	1.77, 0.10	1.30, 0.21	0.49, 0.63	0.97, 0.35
rMSSD	22.5±10.9	23.8±12.4		16.2±7.7	16.4±6.4	-0.43, 0.67	-0.07, 0.95	2.70, 0.02	2.97, 0.01
pNN50	2.7±3.5	2.8±4.3		1.3±1.5	0.7±0.9	-0.06, 0.96	1.26, 0.23	1.98, 0.07	2.02, 0.06
Frequency-domain									
TOTPWR	51.79±14.37	36.50±12.35		41.42±16.78	26.41±12.16	**5.29, 0.0001**	**4.10, 0.001**	2.89, 0.01	**3.43, 0.004**
VLF	51.61±14.28	36.41±12.30		41.28±16.70	26.35±12.13	**5.27, 0.0001**	**4.09, 0.001**	2.90, 0.011	**3.43, 0.004**
LF	0.168±0.126	0.087±0.080		0.146±0.117	0.062±0.070	**3.28, 0.005**	2.22, 0.04	0.61, 0.55	1.12, 0.28
HF	0.037±0.037	0.026±0.027		0.020±0.018	0.013±0.010	1.18, 0.26	1.56, 0.14	2.26, 0.04	2.55, 0.02
LF/HF	5.51±2.82	3.78±1.93		7.31±3.69	5.92±9.71	2.24, 0.04	0.5, 0.62	-2.00, 0.06	-1.02, 0.32
Nonlinear complexity									
MSE	9.54±1.56	11.50±1.85		8.00±1.41	10.18±1.91	**-3.97, 0.001**	**-3.61, 0.003**	**4.88, 0.0002**	1.97, 0.07
MFE	11.70±1.64	13.61±1.90		10.00±1.64	12.39±1.84	**-3.98, 0.001**	**-3.71, 0.002**	**4.85, 0.0002**	1.79, 0.09

The unit of AVNN, SDNN, rMSSD, and pNN50 is millisecond (msec) and the unit of the TOTPWR, VLF, LF, and HF is s^2^/Hz. *Note*. PP = pre-performance period; P = performance period; AVNN = the averages of NN intervals; SDNN = standard deviation of all NN intervals; rMSSD = square root of the mean of the squares of the differences between adjacent NN intervals; pNN50 = percentage of differences between adjacent NN intervals; TOTPWR = total NN interval spectral power; VLF = power in the very low frequency band (0.003–0.04 Hz); LF = power in the low frequency band (0.04–0.15 Hz); HF = power in the high frequency band (0.15–0.4 Hz); LF/HF = ratio between the power of the LF and HF bands; MSE = multiscale sample entropy; MFE = multiscale fuzzy entropy.

**Bold** indicates *p* < 0.005 (based on Bonferroni correction with a selection of 5% significant level among 11 metrics).

## Results

### Time, frequency and nonlinear metrics

**Fig 4:**
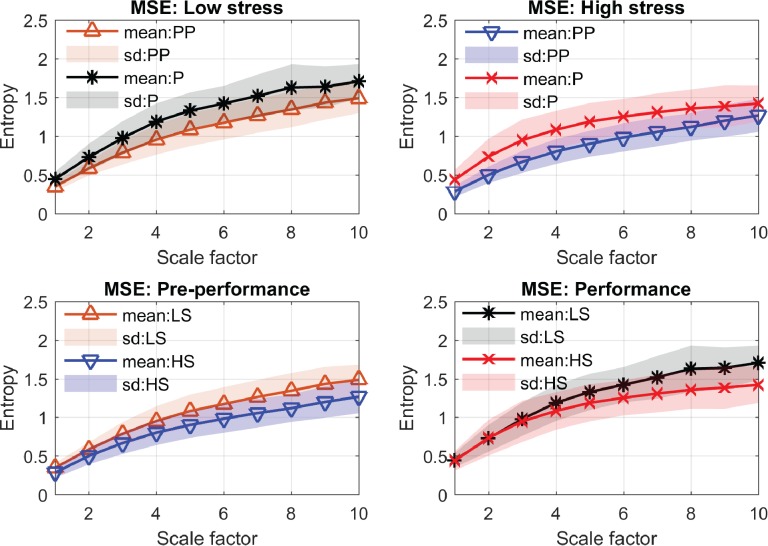
MSE complexity profiles. LS and HS are abbreviations for the low- and high-stress conditions; PP and P are abbreviations for the pre-performance and performance periods, respectively. Note that only the low- and high-stress entropies for the performance period (bottom right) are not statistically different (*p*-value = 0.07).

**Fig 5:**
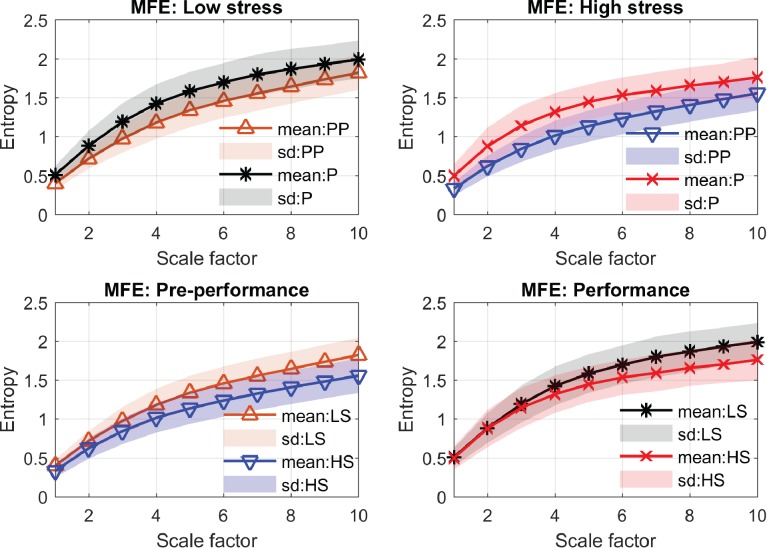
MFE complexity profiles. LS and HS are abbreviations for the low- and high-stress conditions; PP and P are abbreviations for the pre-performance and performance periods, respectively. Note that only the low- and high-stress entropies for the performance period (bottom right) are not statistically different (*p*-value = 0.09).

Table 1 shows that the average and the root mean square of the HRV signals (AVNN and rMMSD) from the low-stress condition (performed without judges) were higher than those from the high-stress condition (performed in front of three judges); in other words, the cardiovascular reactivity of the participants was pronounced in the high-stress condition. However, none of the time-domain metrics shows significant differences in any of the four comparisons except the AVNN metric, which shows discrimination for the performance period where the low- and high-stress conditions were compared. In the frequency domain, the mean power of all frequency bands (TOTPWR, VLF, LF, and HF) showed decreases from the pre-performance to the performance periods, and from the low- to high-stress conditions. The statistical differences between three comparative scenarios (i.e. two periods of performances and the high- vs low-stress in the performance period) were significant for the TOTPWR and VLF metrics. However, the LF metric indicates statistical discrimination only between the two performance periods, while the HF and the LF/HF ratio metrics indicate no statistical discrimination for any of the comparisons. In the nonlinear domain, both MSE and MFE indicated an increase in entropy values from the pre-performance to performance periods, and from the low- to high-stress condition. Statistical comparisons revealed significant differences between most cases for both MSE and MFE, except for the comparison of the low- and high-stress conditions for the performance period. The complexity profiles of the four comparisons using MSE and MFE are shown in Fig 4 and Fig 5.

### Differences between the normalized LF and HF powers

**Fig 6:**
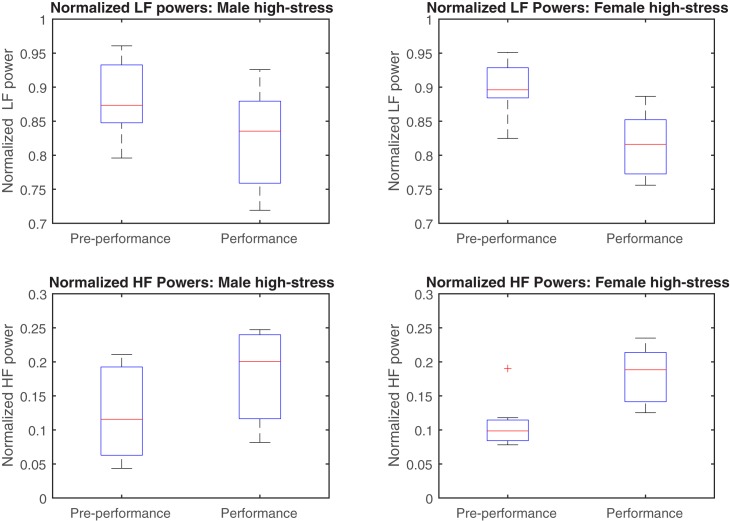
**Box plots of the normalized LF powers from male participants (top left), normalized HF powers from male participants (bottom left), normalized LF powers from female participants (top right), and normalized HF powers from female participants (bottom right).** The red horizontal marker indicates the median.

Analyses of the normalized LF and HF powers revealed differences in the responses of the 9 male and 7 female participants in the high-stress condition. Using a significance level of *p* < 0.05, the t-tests revealed statistically significant decreases in the proportion of LF power from the pre-performance to the performance period in both the male (t_8_ = 2.7, *p* = 0.03) and female participants (t_6_ = 3.0, *p* = 0.02). However, the increase in the proportion of HF power from the pre-performance to the performance period was statistically significant only for the female participants (t_6_ = -2.8, *p* = 0.03); the corresponding *p-*value from the male participants was not significant (t_8_ = -2.2, *p* = 0.06). Significant differences were not found among the results from the low-stress condition. These findings are illustrated in Fig 6, which shows box-plots of the normalized LF and HF powers from the male and female participants during the high-stress condition, where the red horizontal marker indicates the median.

### State anxiety

The reported state anxiety of the musicians was significantly higher in the high-stress condition (mean = 39.12±7.04) than in the low-stress condition (mean = 34.37±7.88) t_15_ = -2.594, *p-*value < 0.05, confirming that the high-stress condition was indeed experienced by the participants as more anxiety provoking than the low-stress condition.

## Discussion and conclusions

We have examined the cardiovascular reactivity of musicians experiencing low- and high-stress performance conditions within the framework of complexity loss theory, quantified using the multiscale entropy (MSE) and multiscale fuzzy entropy (MFE) algorithms. Unlike standard questionnaire-based anxiety assessments, this has been achieved through a suite of objective stress measures based on physiological responses to stress in two scenarios, low- versus high- and before- versus during-performance. Advanced signal processing algorithms for R-peak detection and HRV extraction have been employed to deal with noisy cardiac data in real-life scenarios, while state-of-the-art data analysis techniques in the time, frequency and nonlinear complexity domains have been used to quantify the signatures in HRV related to stress in performance. The analysis has also revealed that currently used spectral analyses of HRV may be inadequate for detecting stress reactivity, as exemplified by the statistically non-significant findings reported in Table 1.

The time-domain analysis based on the AVNN and rMMSD metrics has suggested that, in high-stress conditions, the heart rates of the participants were higher than in low-stress conditions. However, these higher heart rates were accompanied by a smaller difference in heart rate variability when comparing the pre-performance and performance periods, in all time-domain metrics. The standard HRV frequency analysis showed decreases in the LF and HF powers from the pre-performance to performance period, and from the low- to high-stress condition, suggesting a shift of vagal activity from the HF to LF band [23–28] or, in other words, a signature of both SNS and PNS in the LF band [17–22]. This has revealed higher stress in the pre-performance period than during the performance.

The analysis of the normalized LF and HF powers from the pre-performance to the performance period has also revealed a significant decrease in the proportion of LF power, accompanied by a significant increase in the proportion of HF power among the female participants in the high-stress condition. Therefore, assuming a low proportion of HF power to be indicative of low parasympathetic tone, these results suggest that the female participants experienced a more pronounced activation of their parasympathetic nervous systems from the pre-performance to the performance period. This finding has been alluded to in several other studies, with Kattimani [42] reporting that the female stress response is parasympathetically driven and has been confirmed by our results. This disparity between male and female stress responses has been linked to the effect of oestrogen on the autonomic nervous system. A review by Saleh and Connell [43] concluded that oestrogen attenuates sympathetic activity; nevertheless, the exact mechanisms by which oestrogen affects the autonomic nervous system are not fully known.

Our nonlinear analyses have shown that the MSE and MFE approaches have achieved robust discrimination of the underlying features related to the dynamics of the heart, regulated by the autonomic nervous system. Based on the complexity loss theory, both MSE and MFE have shown that the transitions from pre-performance to performance corresponds to a lowering of stress levels in the musicians. The same complexity pattern was presented in the discrimination from the high-stress condition to the low-stress condition. For rigor, these *objective* stress metrics have been benchmarked against the *subjective* state anxiety scores, where the low-stress and high-stress conditions corresponded respectively to lower and higher anxiety reported by the musicians.

The well-documented lack of suitable data acquisition devices (robust to musicians’ movement and motion artefacts, unobtrusive, discreet and comfortable) and a shortage of signal processing algorithms for real-world wearable applications have so far been prohibitive to larger-scale studies of stress experiences in human performance. In this study, we have used both wearable and stationary physiological recording devices and have addressed the imperfections and artefacts in such real-world data through advanced data analysis methods. Our study has focused on combining physiological and psychological measures, analyzed within the framework of the complexity loss theory, to analyze data from a number of performers and to extend a previous single-person study, to address a more general issue of musicians’ emotional and physiological adaptability to psychosocial stressors. However, our study still has limitations, such as: (1). the relatively low sample size, which is due to the constraints of recruiting musicians who were willing and able to provide multiple, polished performances of challenging repertoire and (2) the lack of resting state (baseline) and post-performance cardiographic data, as the study was designed to compare only stress reactivity in pre-performance and performance periods.

Subsequent work will consider joint analysis of multivariate physiological data, such as HRV, respiration rate and skin conductance. The collection, analysis and examination of multivariate data, in relation to strategies for managing stress and enhancing performance quality, promises to offer personally and professionally significant advancements in musicians’ training and skill development, particularly if targeted at the the sensitive period before performance, as shown in this study, and employed in a range of performance contexts.

## Ethics statement

The research was granted ethical approval by the Conservatoires UK Research Ethics Committee and was conducted according to ethical guidelines of the British Psychological Society. Written informed consent was obtained from all participants.

## Supporting information

S1 File Multiscale sample entropy (MSE) and multiscale fuzzy entropy (MFE) algorithms. (DOCX)

S2 File R-R intervals and annotations data of 16 participants in two performance conditions: high stress and low stress. The ZIP folder consists of 16 Matlab files (.mat) and one readme.txt file.
(ZIP)
