# Supplementary material for: Stage call: Cardiovascular reactivity to audition stress in musicians
Source: PLoS One. 2017 Apr 24;12(4):e0176023. doi: 10.1371/journal.pone.0176023 (PMC5402972; doi:10.1371/journal.pone.0176023)
Supplement: S1 File — (DOCX) [file pone.0176023.s001.docx]

**Stage Call: Cardiovascular Reactivity to**

**Audition Stress in Musicians**

Theerasak Chanwimalueang, Lisa Aufegger, Tricia Adjei, David Wasley,

Cinzia Cruder, Danilo P. Mandic* & Aaron Williamon*

* Corresponding authors:

E-mail: d.mandic@imperial.ac.uk (DPM)

E-mail: aaron.williamon@rcm.ac.uk (AW)

**Supporting information**

**S1. Multiscale sample entropy (MSE) and Multiscale fuzzy entropy (MFE) algorithms**

**MSE algorithm**

The MSE algorithm can be described in 3 main steps: (1) coarse graining process, (2) delay vector construction, (3) sample entropy estimation.

**(1) Coarse graining proces*s***

Costa *et al*. [26] defined the coarse graining process as a tool to access a time series at multiple temporal scales. Each scale ϵ is generated by averaging the data points in consecutive non-overlapping windows. For a time series $x\left( i \right)=\{x_{1},x_{2}, x_{3},.., x_{N}\}$, a coarse-grained scale $y_{j}^{(\epsilon)}$ is defined as

$y_{j}^{(\epsilon)}=\frac{1}{\in} \sum_{i=\left( j-1 \right)\epsilon+1}^{j\epsilon} x_{i}$ (1)

where $\epsilon$ represents the scale factor $1\leq j\leq N/\epsilon$. The length of each scale is $N/\epsilon$, for $\epsilon$ = 1, the scaled $y_{j}^{(1)}$ is equal to the original time series.

**(2) Delay vectors construction**

For an embedding dimension $m$, template vectors of length $m$, so-called delay vectors, are defined as

$\boldsymbol{x}_{m}\left( i \right)=\{x_{i}, x_{i+1}, x_{i+2}, \ldots, x_{i+m-1}\}$ (2)

Note all coarse graining scales $y^{(\epsilon)}$ are constructed based on the delay vector $\boldsymbol{x}_{m}$.

**(3) Sample entropy estimation**

Sample entropy represents the conditional probability that within a specified tolerance level, similar patterns found in pairwise considered delay vectors of length $m$, will remain similar when the embedding dimension is incremented to $(m+1)$. For a given tolerance $r$ and embedding dimension $m$, a similar pattern occurs when a maximum distance $d_{i,j}$ of each pairwise consideration of delay vectors fulfills the criterion $d\left[ x_{m}\left( i \right), x_{m}\left( j \right) \right]\leq r, i\neq j$. In other words, the Euclidian distance between the two delay vectors is within a prescribed tolerance level. This results in a number of similar patterns for the whole time series, denoted by $P_{i}$, and the empirical probability for a given pattern (with $m$ the embedding dimension) is defined by $B_{i}^{m}\left( r \right)=\frac{1}{N-n-1}P_{i}$. The total average of similar patterns can therefore be written as

$B^{m}\left( r \right)= \frac{1}{N-n}\sum_{i=1}^{N-m} B_{i}^{m}(r)$ (3)

For the embedding dimensions $m$ and $m+1$, the sample entropy is defined as the negative natural logarithm of the ratio between $B^{m}\left( r \right)$ and $B^{m+1}\left( r \right)$, that is

$SampEnt\left( m,\tau,r,N \right)=-ln[\frac{B^{m+1}\left( r \right)}{B^{m}\left( r \right)}]$ (4)

For multiscale sample entropy, a sample entropy value at each scale ϵ is computed as

$MSE\left( m,\tau,r,N,\epsilon\right)=-ln[\frac{B_{(\epsilon)}^{m+1}\left( r \right)}{B_{(\epsilon)}^{m}\left( r \right)}]$ (5)

where $B_{(\epsilon)}^{m+1}\left( r \right)$ and $B_{(\epsilon)}^{m}\left( r \right)$ are the probabilities of similar patterns found in the delay vectors constructed from the coarse graining scale ϵ.

**MFE algorithm**

In fuzzy entropy, two steps in sample entropy are modified: (1) delay vector construction and (2) fuzzy entropy estimation. A fuzzy membership function is applied to the distance between pairwise delay vectors, instead of directly comparing the distance to the tolerance$r$ as in the sample entropy algorithm. In this way, fuzzy entropy yields a high consistency of entropy values for short time series.

**(1) Delay vector construction**

For an embedding dimension $m$, each delay vector is constructed as in (2) and then subtracted from its local mean $x_{i}^{0}$, to give

$\boldsymbol{x}_{m}\left( i \right)=\left\{ x_{i}, x_{i+1}, x_{i+2}, \ldots, x_{i+m-1} \right\}-x_{i}^{0}$ (6)

where $x_{i}^{0}= \frac{1}{m}\sum_{j=0}^{m-1} x_{i+j}$.

**(2) Fuzzy entropy estimation**

For a given tolerance level $r$, the number of similar patterns is calculated from the Gaussian function

$P_{i}\left( d_{i.j},r \right)=e^{\frac{-d_{i.j}}{r}}$ (7)

After computing $P_{i},$the remaining steps to estimate fuzzy entropy and its multiscale version can be computed from (3) – (5).
